# Unexpected eosinophilia in children affected by hydrocephalus accompanied with shunt infection

**DOI:** 10.1007/s00381-018-3908-5

**Published:** 2018-07-21

**Authors:** Bartosz Polis, Lech Polis, Krzysztof Zeman, Jarosław Paśnik, Emilia Nowosławska

**Affiliations:** 1 Department of Neurosurgery, Polish Mother’s Memorial Hospital Research Institute, Rzgowska st 281/289, 93-338 Łódź, Poland; 2 Department of Pediatrics and Immunology with Nephrology Unit, Polish Mother’s Memorial Hospital Research Institute, Rzgowska st 281/289, 93-338 Łódź, Poland

**Keywords:** *Staphylococcus epidermidis*, Shunt infection, Cerebrospinal fluid (CSF)

## Abstract

**Purpose:**

The aim of the article is to describe an immunological reaction to shunt infection in children with hydrocephalus. The main cause of shunt infection involves methicillin resistant *Staphylococcus epidermidis* (Bhatia et al. Indian J Med Microbiol 35:120–123, 2017; Hayhurst et al. Childs Nerv Syst 24:557–562, 2008; Martínez-Lage et al. Childs Nerv Syst 26: 1795–1798, 2010; Simon et al. PLoS One, 2014; Snowden et al. PLoS One 8:e84089, 2013; Turgut et al. Pediatr Neurosurg 41:131–136, 2005), a bacterial strain which is responsible for the formation of biofilm on contaminated catheters (Snowden et al. PLoS One 8:e84089, 2013; Stevens et al. Br J of Neurosurg 26: 792–797, 2012).

**Methods:**

The study group involved 30 children with congenital hydrocephalus after shunt system implantation, whose procedures were complicated by *S*. *epidermidis* implant infection. Thirty children with congenital hydrocephalus awaiting their first-time shunt implantation formed the control group. The level of eosinophils in peripheral blood was assessed in both groups. Cerebrospinal fluid (CSF) was examined for protein level, pleocytosis, interleukins, CCL26/Eotaxin-3, IL-5, IL-6, CCL11/Eotaxin-1, CCL3/MIP-1a, and MBP. Three measurements were performed in the study group. The first measurement was obtained at the time of shunt infection diagnosis, the second one at the time of the first sterile shunt, and the third one at the time of shunt reimplantation. In the control group, blood and CSF samples were taken once, at the time of shunt implantation.

**Results:**

In the clinical material, the highest values of eosinophils in peripheral blood and CSF pleocytosis were observed in the second measurement. It was accompanied by an increase in the majority of analyzed CSF interleukins.

**Conclusion:**

CSF pleocytosis observed in the study group shortly after CSF sterilization is presumably related to an allergic reaction to *Staphylococcus epidermidis*, the causative agent of ventriculoperitoneal shunt infection.

## Introduction

Methicillin-resistant *Staphylococcus epidermidis* is the main cause of ventriculoperitoneal shunt infection [3, 8, 11, 18, 19, 23]. *Staphylococcus aureus* and *Staphylococcus epidermidis* are responsible for the formation of biofilm on contaminated catheters [19, 21]. A biofilm is a structured community of bacterial cells entrapped in self-produced exopolysaccharide that is attached to an animate or inanimate surface [21]. Biofilms are resistant to antibiotic therapy [19, 21]. Polysaccharide intercellular adhesion (PIA) protects bacterial cells against phagocytosis by macrophages or the action of immunopeptides, but it might also trigger an immunological reaction in the central nervous system (CNS) or peritoneum [21]. The biofilm is responsible for culture-negative cerebrospinal fluid (CSF) in patients with bacterium-contaminated catheters [21]. Biofilms are involved in interactions between the infectious agent, the foreign body, and the immunological environment protecting the infectious agent against the host’s immunological reaction. The principal element of the biofilm is PIA, which helps produce a multilayer community of bacterial cells in the biofilm matrix [21]. Immunological models seeking to elucidate the immune reaction to *Staphylococcus epidermidis* or *Staphylococcus aureus* infections are based on animal models. It is uncertain whether animal models fully depicted the corresponding reactions in humans [7]. Furthermore, some experimental work on animals points to non-compatibility of the immunological reaction between different races of rodents [24].

## Aim

The aim of the article was to describe an immunological reaction to shunt infections in children, particularly the eosinophilic reaction. Elevated levels of eosinophils may be indicative of a latent infection or a hypersensitivity reaction to the shunt material. Inert and non-immunogenic serum proteins may adhere to shunt surfaces. This protein-silicone complex can act as an opsonin, thus stimulating an inflammatory response [17, 19].

## Material and methods

The material includes 30 pediatric patients admitted and operated in the Polish Mother Memorial Hospital, Łódź, Poland.

The study covered 30 pediatric patients (16 male and 14 female patients, gender ratio M/F = 1.14:1). Average age of patients was 8.4 months (± 5.8). The children were hospitalized in the Polish Mother Memorial Hospital between 2010 and 2014. All children from the study group suffered from congenital hydrocephalus. The children were admitted to the hospital because of their first shunt dysfunction secondary to shunt infection. Only children with *S*. *epidermidis* CSF infection were included in the study group.

The control group was composed of 30 pediatric patients who underwent first-time ventriculoperitoneal shunt (VPS) implantation. Children who suffered from hydrocephalus due to inflammation, hemorrhage, or tumor were excluded. Additionally, all patients who could have been treated with neuroendoscopic techniques at any point of the therapy were excluded from the control group. Patient characteristics were as follows: 17 male and 13 female patients, gender ratio M/F = 1.21:1, average age 1.2 months (± 0.3).

CSF analysis in the study subjects and controls included pleocytosis, protein levels, – CCL26/Eotaxin-3, CCL11/Eotaxin-1, IL-8, IL-5, IL-6, and MBP. Additionally, full blood counts were evaluated in both groups to assess absolute and relative changes in eosinophil levels accompanying changes in CSF chemokines.

In the study group, CSF and blood samples were obtained three times.

The first CSF samples were collected at admission (measurement I). After confirmation of infection, all patients were operated to replace VPS by external intraventricular drainage. Proper targeted antibiotic therapy was implemented, which was in every case 5 mg of vancomycin administered every 24 h intraventricularly. The average duration of antibiotic therapy was 20.2 days (SD ± 7.9 days).The first sterile CSF culture specimen was taken into consideration as the measurement II. The sterility of the CSF fluid was confirmed by two additional cultures. The antibiotic therapy was maintained until VPS reimplantation. The average time needed for normalization of CSF cytosis and protein levels was 23.8 days (SD ± 5.27 days). The third CSF sample for research purposes was taken during VPS reimplantation (measurement III).

All 30 pediatric patients from the control group had CSF and blood samples taken at the time of VSP implantation (measurement control).

For safety reasons, only one 5-ml CSF sample from the ventricular system was taken both in the study group and controls.

All CSF specimens were stored at a temperature of − 80 °C. Prior to testing, samples were heated to − 20 °C and then to room temperature. To remove cellular components, the CSF samples were centrifuged at 2000 rpm for 10 min. The supernatant was drawn off in amount of 2.0 ml. The following quantitative determinations were performed using the ELISA assay. Ready-made standardized sets were used (R&D Systems, Inc.; 614 McKinley Place NE; Minneapolis, MN, 55413, USA (Enzyme Immunoassay)). Absorbance reading of the test samples was performed using a microplate DS2 Dynex Technologies, Inc., USA reader. The test is performed using cell culture supernatants. This method is more sensitive compared to the determination in blood serum. Physiological saline was used as negative control.

Absorbance values for each sample were read at the wavelength of 450 nm. Concentration values of the test samples were calculated automatically from the standard curve. Measurements in the following material were made, where the sensitivity of the test, i.e., the minimal detectable concentration, was 0.31–0.20 ng/ml.

In the study group, CSF parameters, such as the levels of – CCL26/Eotaxin-3, CCL11/Eotaxin-1, IL-8, IL-5, IL-6, MBP, cytosis, and protein obtained in three measurements, were compared using variance analysis. Additionally blood morphology was checked at the same time as CSF analysis. Bonferroni post hoc analysis was used to test for significant differences between the three measurements.

The above parameters obtained in each of the three measurements in the study group were compared with the corresponding parameters in the control group using the Friedman test (post hoc Wilcoxon test with Bonferroni correction for the number of comparisons). The Kolmogorov-Smirnov test with Lilliefors correction was used to assess the normality of distribution. The homogeneity of variances was tested with the Mauchly test. For non-homogenous variances, the Greenhouse–Geisser correction was applied. The level of statistical significance was set at *p* < 0.05. SPSS 17.0 software was used for the entire statistical analysis.

## Results

**Fig. 1:**
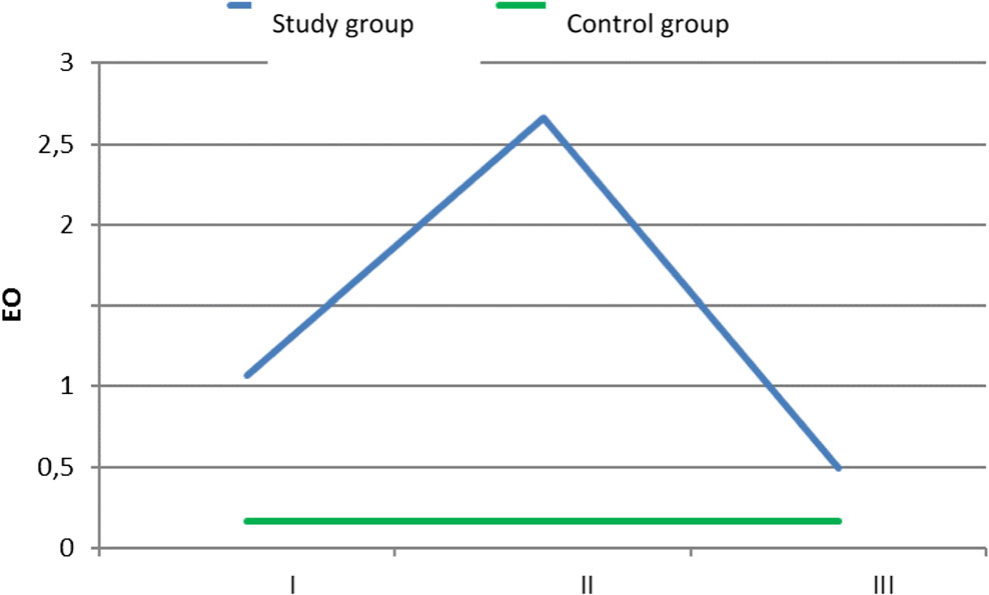
The average level of eosinophils × 10^3^ c/μl in the control group and in the study group (I—in the first measurement, II—in the second measurement, III—in the third measurement)

The most striking relationship observed in the presented material was the increase in eosinophils in the blood at the time of the second measurement. The average result was 2.66 × 10^3^ c/μl (± 0.67 × 10^3^ c/μl). Variance analysis (*p* < 0.001) showed that it was significantly higher in comparison with the first measurement, in which the average level of eosinophils was 1.07 × 10^3^ c/μl (± 0.12 × 10^3^ c/μl). Finally, variance analysis (*p* < 0.001) indicated that the average eosinophil level measured shortly before shunt implantation was significantly lower compared to the result obtained at the time of the diagnosis of infection 0.49 × 10^3^ c/μl (± 0.12 × 10^3^ c/μl). In the control group, the level of eosinophils was significantly lower than that in the study group—0.17 × 10^3^ c/μl (± 0.06 × 10^3^ c/μl) (*p* < 0.001) in each measurement (Fig. 1).

**Fig. 2:**
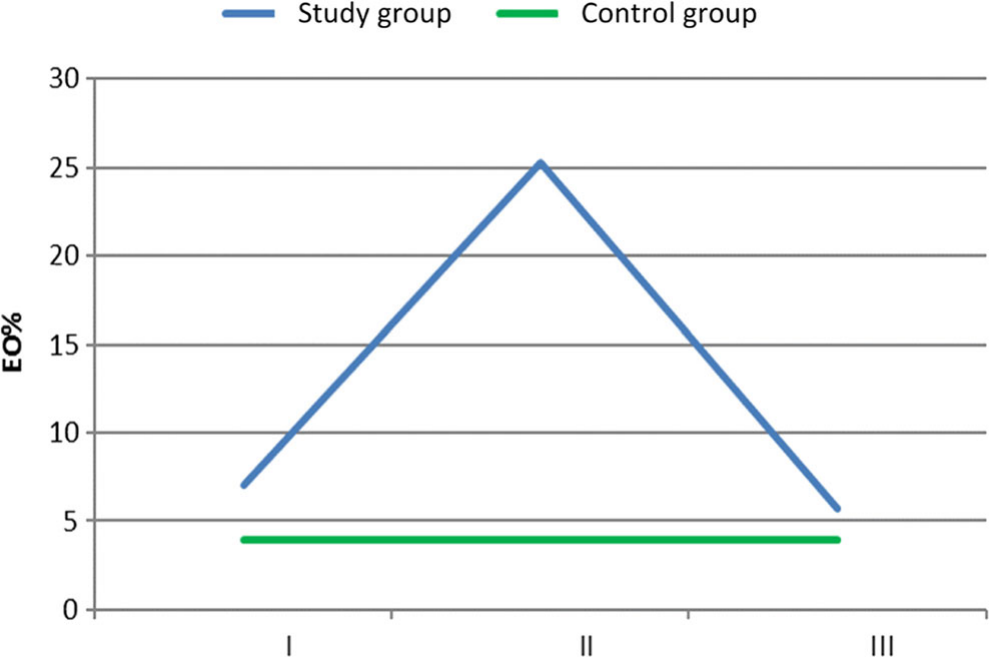
The average percentage of eosinophils in blood cell differential count in controls and the study group at individual measurements (I—the first measurement, II—the second measurement, III—the third measurement)

The same relationships between particular measurements took place in the blood cell pattern. The average percentage of eosinophils reached the highest level at the second measurement. It was 25.25% (± 6.30%).The difference between it and the rest of measurements was of statistical significance (*p* < 0.001). The lowest level of eosinophil percentage was reached in the third measurement. The average percentage was 5.70% (± 0.67%). It was significantly lower than that in the first measurement—7.01% (± 0.69%) (*p* < 0.001). In the control group, eosinophil percentage measured in the blood just before shunt implantation was significantly lower than that in all measurements in the study group (*p* < 0.001). It was on average 3.89% (± 0.92%) (Fig. 2).

**Fig. 3:**
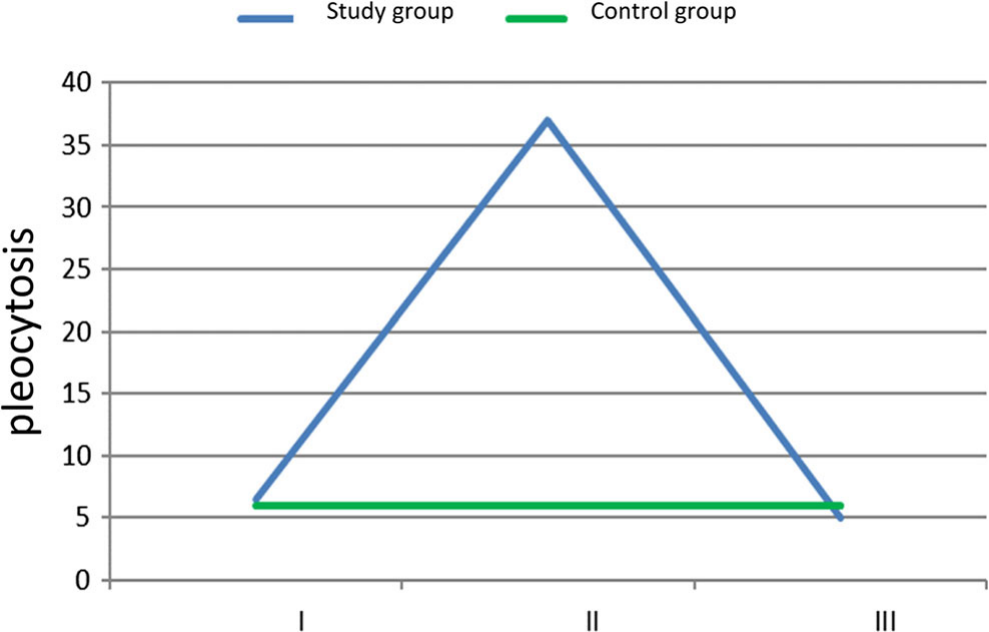
Median CSF pleocytosis (c/μl) in controls and the study group (I—the first measurement, II—the second measurement, III—the third measurement)

CSF pleocytosis revealed a similar relationship between individual measurements as the one seen for eosinophils. The highest level of pleocytosis was reached in the second measurement. The average result was 106.03 c/μl (± 145.21 c/μl). It was higher than that in the first measurement 7.90 c/μl (± 7.80 c/μl) and the third measurement 13.10 c/μl (± 19.53 c/μl). Both relationships were statistically significant according to variance analysis test (*p* < 0.001). However, unlike the eosinophils, there was no statistically relevant difference between the first and the third measurements (*p* > 0.999). In the control group, pleocytosis was significantly lower than that in the second measurement according to the Mann–Whitney *U* test (*p* = 0.001), with an average value of 7.83 c/μl (± 6.81 c/μl). Also, in contrast to eosinophils, there were no statistically significant differences between the control group and the study group in the first and in the third measurements. Changes in median pleocytosis in the study group are shown in Fig. 3.

**Fig. 4:**
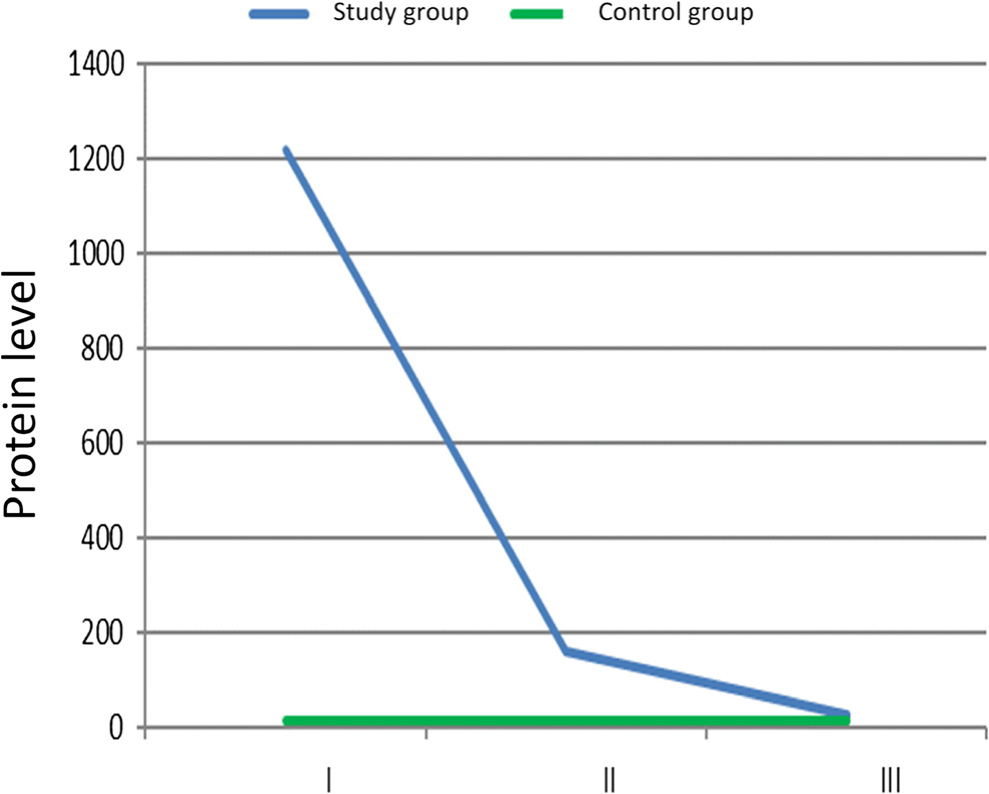
Median CSF protein level (mg%) in controls and the study group (I—the first measurement, II—the second measurement, III—the third measurement)

The opposite trends were observed in the analysis of total CSF protein level. The highest level was already reached in the first measurement. It was on average 1291.63 mg% (± 497.80 mg%). In subsequent measurements, the average protein level was significantly lower. The second one was 179.83 mg% (± 83.12 mg%), *p* < 0.001. The lowest protein level in the study group was found in the third measurement, and it was on average 35.30 mg% (± 25.20 mg%), *p* < 0.001. According to the Mann–Whitney *U* test, CSF protein level was significantly lower in controls than that in the study group. It was on average 17.47 mg% (± 9.24 mg%). This value was lower in comparison with measurements I (*p* < 0.001), II (*p* < 0.001), and III (*p* = 0.013). Changes in median CSF protein levels in the study group in individual measurements are shown in Fig. 4.

## Discussion

Shunt infection may be associated with the number of shunt revisions, patient age, manual shunt system, and exposure to surgical instruments. The overall incidence amounts to 3–15% of all implanted shunt systems [2, 8, 12, 14, 20, 21]. Ninety percent of all microorganisms infecting CSF shunting devices belong to *Staphylococcus* and *Streptococcus* species [1, 17]. The clinical material was notable for an increase in pleocytosis from the very beginning of known infection, in which the dysfunction of infected shunt was present [5, 17, 22]. This observation is totally at odds with the results described in the present article. Indeed, no visible increase in pleocytosis was not observed in the study group. Eosinophilia may be present very often in bacterial infection [17, 19, 22]. In the study group, the highest level of eosinophils in peripheral blood was observed at the time of the second measurement (Fig. 1). The same tendency was observed in the analysis of blood cell differential count (Fig. 2). It is possible that the increase in eosinophils in the study group could account for the highest CSF pleocytosis values observed in the second measurement (Fig. 3). Eosinophilia in CSF was defined as ≥ 5% or according to other authors ≥ 10% eosinophils in the CSF [5, 17, 22] Eosinophils in the CSF are not particularly specific for bacterial encephalitis, which is more likely to be associated with CSF neutrophilia [10]. This reaction could be responsible for an early increase in CSF protein levels observed in the study group (Fig. 4). Eosinophilic encephalitis is more typical for parasitic infections, e.g., *Angiostrongyliasis cantonensis* (~ 70% of patients) and *Gnathostoma spinigerum* (> 55% of patients). Other infections with a concomitant eosinophilic reaction may include different microorganisms, such as helminths, fungi, bacteria, and viruses [6, 11, 22]. An allergic reaction could be another causative agent in eosinophilic encephalitis. The main reason for an increase in CSF eosinophil levels after shunt implantation is still unknown. The increase in eosinophils could be present when the CSF was free from bacterial contamination or in the absence of shunt dysfunction [17]. It is surprising that in the presented material, the peak level of eosinophils in peripheral blood was reached after microbial elimination. A possible explanation could be related to the ability of *Staphylococcus* species to form the so-called biofilms [21]. This structure is responsible for interaction with the immunological environment. Therefore, it is possible that despite the absence of bacterial strains in the CSF, they are still present on the surface of intraventricular catheters [21]. The whole process for the migration and activation of eosinophils in patients with ventriculoperitoneal shunts as a result of either infection or allergy is fully described [17]. As it has been mentioned at the beginning of the article, in contrast to neutrophils and monocytes, eosinophils have an ability to adhere well to shunt catheters [22]. That ability could play an important role in the case of intraventricular catheter contamination by *Staphylococcus* species [17, 19]. Another explanation for eosinophilic activation may be shunt obstruction accompanying shunt infection [17]. In addition, residual CSF in the chamber of the occluded shunt system could trigger an allergic reaction [5]. Unfortunately, this process does not explain the culmination of eosinophilia in peripheral blood at second measurement, because at that time, infected shunts were removed. This procedure is consistent with the established operative protocol in the context of shunt infections and is performed by many clinicians [1, 2, 20–23]. The third presumed cause of an allergic reaction to shunt infection could be reaction to intraventriculary administered antibiotic therapy [1, 19]. Particularly, vancomycin overdose could be responsible for the eosinophilic reaction [1]. The authors agree that maximal therapeutic effectiveness could be achieved when vancomycin concentrations in CSF reach the level of 5–10 μg/mL. CSF output from a ventricular system could influence the concentration of administered vancomycin. CSF is completely exchanged three to four times per day on average. Patients with ventriculitis may have decreased clearance of vancomycin secondary to a fall in CSF production. Theoretically, CSF vancomycin concentrations < 5 and > 10 μg/ml are out of therapeutic window [1]. In the presented clinical material, Vancocin was intraventricularly administered according to the same time dose regimens up to the shunt implantation, so there is no explanation to decrease of the level of eosinophils just before shunt implantation [17]. The significant differences in the level of eosinophils in blood between the control and study groups could be an allergic reaction to implanted artificial material like intraventricular catheters (Figs. 1 and 2). Eosinophilia as an allergic reaction could be disclosed relatively late 8 months after shunt implantation [4, 22]. The control group is free from the allergic reaction due to shunt implantation. But regarding the role of immunization of implanted material, there is still unknown why the eosinophils decrease in the study group in the third measurement (Figs. 1 and 2). In that group, intraventricular catheter is always present in the lumen of cerebral ventricles and should exert constant stimulation to allergic reaction. An eosinophilia in the peripheral blood could be a reaction to the bleeding during shunt removal too. It is observed in the case of subarachnoid bleeding [4]. But in the study group, patients after bleeding into ventricular system were excluded. In order to understand eosinophilic encephalitis induced by shunt infections, sometimes analogies of other diseases are found [16]. There is no natural presence of such morphotic elements like leucocytes in the CNS because of the blood–brain barrier (BBB) mechanism existence. So the central nervous system is the place of immune privilege [7, 21]. The innate morphotic elements of immunologic reaction in CNS are the following: microglial cells, infiltrating myeloid cells, astrocytes, oligodendrocytes, and NG2+ glia (also termed polydendrocytes or oligodendrocyte progenitor cells) [7, 21]. Microglia progenitors enter the CNS at embryonic day 9.5–10.5 [7, 21]. In the CNS, pro-inflammatory cytokines like IL-1, IL-6, TNF-α, and INF-β are produced by resident cell and are less migrated by lymphocyte. The sources of cytokines are the following: cerebrovascular endothelial cells, microglia, and astrocytes [21]. In the material, it was checked the level of IL-6 in the cerebrospinal fluid. Similarly to eosinophils, the second measurement reached the highest level in the analysis. Contrary to the eosinophilia, the third measurement was the same as that in the control group (Table 1). IL-6 starts in the inflammation process and takes part in the erythropoiesis and many allergic reactions [15]. The role which acts by IL-6 could explain the observed level dynamism in CSF. So it exposes the role of innate morphotic response in the central nervous system. Clinical material presented in the article comprises other elements of the observed humoral immunological response. The next one was IL-5 measured in the cerebrospinal fluid (Table 1). IL-5 could stimulate the production of eosinophils from pluripotent bone marrow stem cells and their release to circulation too. This interleukin is produced by Th2-type helper lymphocytes in response among others to allergies. Mature eosinophils adhere to endothelial cells and migrate into the tissues after exposure to this chemoattractant. So, the level of IL-5 in the cerebrospinal fluid depicted the peripheral immunological response [15, 17]. The elevated level of IL-5 lasts longer in the study group than that of IL-6, because it is a visible increase of this lymphokine through the whole period of observation (Table 1). The role of that agent in the allergic reaction is proved by works describing role of corticosteroids in promotion of ventriculoperitoneal shunt survival by blocking the effect of eosinophil meaning as production, migration, and/or granule release, by inhibiting the production and/or release of IL-5 in the bone marrow, and preventing chemoattractant production and upregulation of adhesion molecules [17]. The assumption that increased level of CSF pleocytosis in the second measurement is connected with the level of eosinophils in the peripheral blood is supported by chemoattractant dynamism in CSF observed in the clinical material (Table 1). The peak level in CSF of receptor for eotaxin (CCL-11), small cytokine belonging to the CC chemokine family, was obtained in the first and second measurements, and it was significantly higher than that in the third measurement and in the control group (Table 1). CCL-11 selectively recruits eosinophils by inducing their chemotaxis. It is a component of allergic response [13]. Another strong chemoattractant analyzed in the clinical material receptor for eotaxin (CCL-26) remained increased in the study group through the whole period observation. It was also significantly higher than that in the control group. It could be possible that it is a natural antagonist for CC chemokine receptors 1 and 5 of human chemokine and acts rather a regulatory role in the observed allergic reaction [13]. Finally, it was examined the level in CSF of CCL-3(chemokine) and MBP (myelin granule protein) both produced by eosinophils [9] (Table 1). The highest level of both substances in CSF was obtained in the study group in the second measurement. The result strongly suggests that observed pleocytosis in CSF could be composed from eosinophils.

**Table 1:** The analyzed lymphokines and proteins in the study group and in controls. *C*, control group, I—measurement I, II—measurement II, III—measurement III

	Measurement I (ng/ml)	Measurement II (ng/ml)	Measurement III (ng/ml)	Control group (ng/ml)	Statistically important differences between individual measurements
CCL26/Eotaxin-3	7.97	6.86	7.59	4.40	C < I= II = III
IL-5	1.51	1.84	1.65	0.56	C < I = II = III
IL-6	320.39	555.05	50.60	46.35	C = III < I < II
CCL11/Eotaxin-1	21.68	16.79	9.64	5.60	C < III < I = II
CCL3/MIP-1a	1.65	27.28	15.60	17.84	II > C > III > I
MBP	0.01	1.60	1.83	0.52	I < = II = (C < III)

## Conclusion

1. CSF pleocytosis observed in the study group shortly after CSF sterilization is presumably connected with an allergic reaction to *Staphylococcus epidermidis* responsible for ventriculoperitoneal shunts infection.
2. The allergic reaction could be also responsible for absolute and relative increase of eosinophils in peripheral blood just after CSF sterilization.
3. Ceasing the allergic reaction to the *Staphylococcus epidermidis* could make shorter process of treatment bacterial infection.
